# B-Cell-Activating Factor and the B-Cell Compartment in HIV/SIV Infection

**DOI:** 10.3389/fimmu.2017.01338

**Published:** 2017-10-27

**Authors:** Gwenoline Borhis, Maria Trovato, Nada Chaoul, Hany M. Ibrahim, Yolande Richard

**Affiliations:** ^1^INSERM u1016, Institut Cochin, Paris, France; ^2^CNRS UMR 8104, Paris, France; ^3^Université Paris-Descartes, Paris, France; ^4^Commissariat à l’Energie Atomique, Institut des maladies Emergentes et Thérapies innovantes, Service d’Immuno-Virologie, Fontenay-aux Roses, France

**Keywords:** B-cell-activating factor, B-cells, dendritic cells, germinal center, HIV, memory B-cells, follicular helper T-cells, SIV

## Abstract

With the goal to design effective HIV vaccines, intensive studies focused on broadly neutralizing antibodies, which arise in a fraction of HIV-infected people. Apart from identifying new vulnerability sites in the viral envelope proteins, these studies have shown that a fraction of these antibodies are produced by self/poly-reactive B-cells. These findings prompted us to revisit the B-cell differentiation and selection process during HIV/SIV infection and to consider B-cells as active players possibly shaping the helper T-cell program within germinal centers (GCs). In this context, we paid a particular attention to B-cell-activating factor (BAFF), a key cytokine in B-cell development and immune response that is overproduced during HIV/SIV infection. As it does in autoimmune diseases, BAFF excess might contribute to the abnormal rescue of self-reactive B-cells at several checkpoints of the B-cell development and impair memory B-cell generation and functions. In this review, we first point out what is known about the functions of BAFF/a proliferation-inducing ligand and their receptors [B-cell maturation, transmembrane activator and CAML interactor (TACI), and BAFF-R], in physiological and pathophysiological settings, in mice and humans. In particular, we highlight recent results on the previously underappreciated regulatory functions of TACI and on the highly regulated production of soluble TACI and BAFF-R that act as decoy receptors. In light of recent data on BAFF, TACI, and BAFF-R, we then revisit the altered phenotypes and functions of B-cell subsets during the acute and chronic phase of HIV/SIV infection. Given the atypical phenotype and reduced functions of memory B-cells in HIV/SIV infection, we particularly discuss the GC reaction, a key checkpoint where self-reactive B-cells are eliminated and pathogen-specific memory B-cells and plasmablasts/cells are generated in physiological settings. Through its capacity to differentially bind and process BAFF-R and TACI on GC B-cells and possibly on follicular helper T-cells, BAFF appears as a key regulator of the physiological GC reaction. Its local excess during HIV/SIV infection could play a key role in B-cell dysregulations.

## Introduction

During pathogenic HIV/SIV infection, efficient antibody (Ab) protection hardly develops whereas immunoglobulin overproduction, germinal center (GC) hyperplasia (1), and increased recruitment of follicular helper T-cells (T_FH_) into GC occur concurrently from the acute phase of infection (2–6). In addition to several reports showing increased proportions of atypical memory B-cells in lymphoid organs and transitional B-cells in blood (7), recent molecular investigations established that a fraction of broadly neutralizing Abs (bNAbs) are produced by self/poly-reactive B-cells (8). In addition to direct B-cell activation by viral envelope proteins, inflammation is thought to play a major role in shaping these changes in B-cell phenotype and in virus-specific Ab responses (9–12). B-cell-activating factor (BAFF)/a proliferation-inducing ligand (APRIL) are instrumental cytokines for B-cell ontogeny and humoral responses in physiological settings (13), while their overproduction is detrimental in numerous autoimmune disorders (14, 15). During HIV/SIV or plasmodium infection, increased BAFF levels occur concurrently with expansion of atypical memory B-cells and inefficient Ab response (16–19). Thus, BAFF was thought to exert detrimental actions on pathogen-specific B-cells, and its overexpression has been associated with HIV/SIV disease progression (20–22). However, BAFF excess favors the expansion of immature-transitional B-cells and promotes self-Abs in mice and in patients with autoimmune diseases (23–25). Through a similar pathway, BAFF might be beneficial in expanding the pool of HIV cross-reactive B-cells, a potential source of bNAbs. Therefore, the role of BAFF excess in generating HIV/SIV-specific memory B-cells and neutralizing Abs needs to be further clarified. In this review, we first summarize what it is known about BAFF/APRIL and their receptors, with a special attention to transmembrane activator and CAML interactor (TACI), which might act as a key regulator of B-cell activation, BAFF-R shedding (26) and possibly self-reactivity. We then highlight data obtained in mice, humans, and macaques with the aim to better appreciate the role of BAFF and its receptors, BAFF-R and TACI, in HIV/SIV progression and in the expansion of HIV/SIV cross-reactive B-cells.

## BAFF/APRIL and Their Receptors

The BAFF belonging to the tumor necrosis factor (TNF) superfamily (also called BLys) was first described as a key regulator of B-cell homeostasis and survival in mice and in humans (13). BAFF exerts its effects by binding to three different receptors: B-cell maturation (BCMA) (27, 28), TACI (29), and BAFF-R/BR3 (BLys receptor 3) (30). A highly similar homolog of BAFF (called APRIL) (31) also binds TACI and BCMA but not BAFF-R (32). APRIL only exists as a soluble form cleaved intracellularly, whereas BAFF can be found in both membrane-bound and soluble forms. In myeloid cells, BAFF is expressed on the cell surface as a membrane-bound form (mBAFF) and can then be released as a soluble form after cleavage by furin protease (33–35). Neutrophils directly release BAFF and APRIL as soluble cytokines (36, 37), whereas plasmacytoid dendritic cells (pDC) are unable to cleave mBAFF into its soluble form (19, 38, 39).

Through different expression and affinity for BAFF and APRIL, BAFF-R, TACI, and BCMA finely tune B-cell ontogeny and immune responses with species specificity (30, 40–44). Functional BAFF-R and TACI are expressed in B1 cells (45), and aging APRIL-transgenic mice develop B1 lymphoma (46, 47), whereas BAFF- and BAFF-R-deficient mice have normal proportions of B1 cells (48) (Table 1). This indicates that the TACI–APRIL pair likely plays a dominant role in murine B1 homeostasis. Absent from early transitional B-cells (T1, CD10^+^CD21^lo^), BAFF-R expression is acquired by transitional type-2 B-cells (T2, CD10^+^CD21^+^), and deficiency in BAFF-R inhibits B-cell ontogeny beyond the T1/T2 transition (49). However, this blockade is not absolute, and small proportions of mature B-cells are still present in BAFF-R-deficient mice and, to a lesser extent, in BAFF-deficient mice that mount residual responses to T-dependent (TD) antigens (50, 51). Consistently, BAFF- or BAFF-R-deficient mice form rudimentary GC in response to TD antigens (52, 53). Absent from naïve and memory B-cells, BCMA is dispensable for the survival of mature B-cells, spleen architecture, and GC development. Response to TD or T-independent (TI) antigens and isotype class switching are normal in BCMA-deficient mice (54–56). However, BCMA is important for long-term plasma cell biology (55, 57, 58) and antigen presentation (59). Upon binding to BCMA, APRIL and, to a lesser extent, BAFF promotes the survival of long-lived plasma cells in bone marrow (55). BCMA therefore constitutes one privileged target for the selective killing of malignant plasma cells, such as multiple myeloma cells (60, 61). Consistent with the recent description of constitutive BCMA shedding from the membrane of plasma cells by a γ-secretase (62), high serum BCMA level correlates with disease status and constitutes a valuable biomarker in multiple myeloma (63). Moreover, TACI expression distinguishes TACI^lo^ from TACI^hi^ myeloma, the latter with a signature of plasma cells, which are more dependent on bone marrow signals (64), likely osteoclast-derived BAFF/APRIL and IL6 (65). Accordingly, TACI^hi^ myelomas are expected to be more responsive to BAFF-related immunotherapies. Based on these data in malignant cells, normal circulating plasmablasts are thought to be TACI^lo^ in contrast to long-lived plasma cells present in bone marrow that would be TACI^hi^.

**Table 1:** Phenotype of B-cell subsets and expression of B-cell-activating factor (BAFF)/a proliferation-inducing ligand (APRIL) receptors.

B-cell subset	Phenotype	BAFF/APRIL receptor expression	Reference
Mouse B1 cells	CD19^hi^SIgM^hi^SIgD^lo^CD43^+^CD1d^int^CD23^−^CD5^+^ (B1a) or CD5^−^ (B1b)	BAFF-R^+^TACI^+^	(45–48)
Early transitional B-cells (T1)	CD19^+^IgM^hi^CD10^+^CD24^hi^CD38^hi^CD21^lo^	BAFF-R^−/lo^TACI^+/−^	(25, 49, 51, 66–69)
Transitional type-2 B-cells (T2)	CD19^+^SIgM^hi^SIgD^lo^CD10^+^CD24^hi^CD38^hi^CD21^+^	BAFF-R^+^TACI^+/−^	
Marginal zone B-cells	CD19^+^CD20^+^IgM^hi^CD21^hi^SIgD^+^CD23^−^CD27^+^	BAFF-R^+^TACI^hi^ (short > long isoform)	(48, 54–56, 66, 70–75)
Naïve follicular B-cells	CD19^+^CD20^+^SIgD^hi^SIgM^+^CD21^+^CD23^+^CD27^−^CD95^−^	BAFF-R^hi^TACI^−/lo^ (long isoform)	(48, 52–55, 56, 70–74)
Germinal center (GC) B-cells centroblasts	CD19^+^CD20^+^CD27^int^Bcl6^+^Ki67^+^Sig^−^CD95^+^CD10^+^CXCR4^+^	BAFF-R^hi^TACI^lo^	(48, 52, 53, 56, 70, 71, 74, 76, 77)
GC B-cells centrocytes	CD19^+^CD20^+^CD95^+^CD10^+^CD38^+^CD83^+^SIgM/A/G^+^	BAFF-R^hi^TACI^+^	(78, 79)
Resting memory	CD19^+^CD20^+^SIgD^−^SIgG/A^+^CD27^+^CD21^+^CD95^+^	BAFF-R^+^TACI^hi^ (short > long isoform)	(48, 69, 72, 73, 80)
Activated memory	CD19^+^CD20^hi^SIgD^−^SIgG/A^+^CD27^+^CD21^lo^CD95^+^	BAFF-R^int^TACI^+^BCMA^+^	(68, 81–83)
Tissue-like memory	CD19^+^CD20^hi^SIgD^−^SIgG/A^+^CD27^−^CD21^lo^CD95^+^	BAFF-R^int^TACI^+^BCMA^+^	
Plasmablasts	CD19^+^CD20^lo^CD21^lo^CD27^hi^CD38^hi^CD138^lo^	BAFF-R^lo^TACI^lo^BCMA^+^	(64, 68, 81)
Plasma cells	CD19^lo^CD20^−^CD27^hi^CD38^+^CD138^hi^	BAFF-R^lo^TACI^hi^BCMA^hi^	(55, 57, 58, 64)

## TACI: A Multifaceted Receptor for BAFF/APRIL in Mice and Humans

### Conventional and Regulatory Functions of TACI in Mice

BAFF-R is expressed by most follicular B-cells whereas TACI is absent (or very low) from naïve B-cells but highly present on marginal zone (MZ) and class-switched memory B-cells (48, 72, 73). TACI-deficient mice fail to respond to type-2 TI antigens (TI-2) but retain normal TD response (Table 2). However, they have reduced serum IgM and IgA levels, but normal IgG levels (48). *In vitro*, Castigli et al. have established that the murine TACI–APRIL pair is mandatory for IgA class switching and plays a dominant role over the BAFF-R–BAFF pair in IgG class switching (56). In another mouse model, TACI deficiency induces hyperplasia, enlarged MZ B-cell pool (66) and lupus-like autoimmune manifestations in aged mice (84). In agreement with TACI controlling exacerbated B-cell activation, knock-in mouse carrying a C76R mutation that impairs TACI-induced NF-κB activation develops splenomegaly with increased proportions of MZ and follicular B-cells (74). So, murine TACI that positively controls response to TI-2 antigens and IgA class switching can also deliver inhibitory signals that dampen abnormal B-cell activation and expansion. *In vitro*, Figgett et al. recently demonstrated that BAFF binding to TACI selectively limits TI innate response of TLR4-activated MZ B-cells by promoting FAS/FASL-mediated apoptosis (75). This process is thought to prevent inappropriate TI B-cell responses such as the expansion of self-reactive B-cells, and therefore to safeguard peripheral immune tolerance. Thus, membrane TACI controls excessive expansion/response of various mouse B-cell subsets.

**Table 2:** Consequences of deficiency in BAFF-R and transmembrane activator and CAML interactor (TACI) in genetically modified mice and common variable immunodeficiency (CVID) patients.

Receptor	Phenotype	Reference
BAFF-R KO mice and A/WySnJ mice	Blockade of B-cell development at the T1/T2 transition Small proportions of marginal zone (MZ) and follicular B-cells Normal proportions of B1 cells Rudimentary GC but rapid involution, residual TD response Impaired class switching	(53, 76)

CVID patients with BAFF-R deficiency	No BAFF-R membrane expression Reduced numbers of mature B-cells, in particular MZ B-cells Expansion of T2 B-cells in blood Substantial numbers of naive and memory B-cells Reduced levels of IgM and IgG but normal IgA levels	(77)

TACI KO mice	Normal MZ and B1 cells Impaired response to TI-2 Ags with low IgM/A levels Normal IgG levels Normal TD responses	(48)

	B-cell lymphoproliferation and enlarged MZ B-cell pool Overproduction of Ig in response to TD Ags Lupus-like autoimmune manifestations in aged mice Increased production of self-reactive antibodies	(66, 84)

CVID patients with TACI deficiency	No or reduced TACI membrane expression Impaired NF-κB signaling Impaired IgA and IgG class switching Reduced response to TI-2 Ags B-cell lymphoproliferations, splenomegaly Increased frequency of autoimmune diseases Lack of lupus-like symptoms	(56, 70, 71)

TACI KI C76R mice	Normal membrane expression of TACI Impaired NF-κB activation Increased proportions of MZ and follicular B-cells Splenomegaly	(74)

### BAFF-R and TACI in Humans: Lessons from Patients with Genetic Immunodeficiency

Spontaneous mutations occurring in individuals or families offer the opportunity to compare the biological importance of key molecules between mice and humans (Table 2). Studies in two patients with common variable immunodeficiency (CVID) carrying a homozygous deletion in BAFF-R gene, that precludes its membrane expression, confirm the key role of BAFF-R in human B-cell development. However, the phenotype of these patients is less severely compromised than that of BAFF-R-deficient mice, with significant numbers of circulating memory B-cells and normal IgA levels, despite B-cell lymphopenia and low levels of circulating IgM and IgG (77).

Similarly, the phenotype of CVID individuals with TACI deficiency differs from that of TACI-deficient mice (70, 71). These individuals combine Ab-deficiency syndrome, B-cell lymphoproliferation, and increased frequency of autoimmune manifestations without symptoms of lupus-like disease. Two homozygous mutations at positions C104R (the human equivalent of murine C76R) and S144X impair class switching to IgA but also to IgG, unlike TACI-deficient mice (71). Whereas TACI was expressed on B-cells from all individuals with heterozygous mutations (including C104R), its signaling was impaired leading to abnormal Ig production *in vitro* (70). Consistent with data in TACI-deficient mice, individuals with TACI deficiency have a strongly reduced response to TI-2 antigens with recurrent infections and more frequently develop splenomegaly. Thus, human TACI is mandatory for response to TI-2 antigens and IgA/G class switching. Splenomegaly and autoimmune manifestations in these patients clearly indicate that TACI also acts as negative regulator of B-cell expansion/response in humans.

Moreover, two recent studies evidenced the release of soluble TACI and BAFF-R, acting as soluble decoy receptors. Surface TACI is constitutively cleaved by ADAM17 from human and murine B-cells, producing a homotrimer acting as a soluble decoy receptor for BAFF and, to a lesser extent, for APRIL. Subsequent cleavage of its remaining membrane-bound C-terminal domain by γ − secretase prevents residual NF-κB activation (85). While ADAM17 cleaves BAFF-R from dark zone GC B-cells (centroblasts), BAFF-R cleavage by ADAM10, which depends on BAFF binding and TACI expression, occurs in memory and MZ B-cells as well as in light zone GC B-cells (centrocytes) (26). By amplifying BAFF-R cleavage from centrocytes, BAFF excess might impair B-cell selection and high affinity Ab maturation. Taken together, these results highlight a previously unexpected role for TACI as a key modulator of BAFF-mediated responses.

A supplementary level of complexity was introduced by the identification of two isoforms of human TACI produced by alternative splicing of the unique encoding gene. One isoform with two extracellular ligand-binding domains resembles murine TACI whereas the second isoform, which contains only one binding domain, was referred to as TACI-short by authors (80). *In vitro* studies have established that TACI-short binds APRIL and BAFF with higher affinity than the other isoform and that its triggering by either ligand leads to a more potent activation of canonical NF-κB pathway (86) and plasma cell differentiation (80). Consistent with previous data (87), intense NF-κB activation downstream TACI-short correlates with enhanced recruitment of MyD88. In particular, messengers of both TACI isoforms were found in isolated resting memory (RM, CD21^+^CD27^+^) and MZ B-cells, with TACI-short mRNA being present in higher amounts (80). It is therefore possible that the response to BAFF/APRIL is finely modulated through binding to TACI trimers containing various ratio of each isoform. Mechanisms favoring preferential TACI-short expression *in vivo* remain to be identified but, *in vitro*, TLR9 ligands strongly upregulate it in CD27^+^ B-cells. To what extent each TACI isoform contributes to the biology of memory B-cells and long-lived plasma cells remains to be studied. Since survival of memory B-cells is less dependent on BAFF *in vivo* than that of transitional and naïve B-cells, TACI-short expression might confer them an exceptional responsiveness to limited BAFF amounts. Whether TACI-short is released and whether it differently modulates BAFF-mediated BAFF-R cleavage on RM B-cells should be examined.

## Evidence for Soluble and Membrane BAFF Overexpression During HIV/SIV Infection

Elevated circulating levels of BAFF and/or APRIL are associated with autoimmune diseases, chronic inflammation (14, 88), or occur after CD20 B-cell depleting therapy (89, 90). Because chronic inflammation and hypergammaglobulinemia are hallmarks of chronic HIV-1 infection, serum BAFF levels were first measured in chronically HIV-infected individuals (91). In this pioneer report, authors observed increased BAFF levels in most individuals, correlating with levels of self-Abs only in individuals with more than 200 CD4 T-cells per microliters. In these individuals, classical monocytes (CD14^hi^) overexpressing mBAFF were identified as a major source of soluble BAFF. Extending these first results, Fontaine et al. have evidenced increased levels of serum BAFF in HIV-infected people, with a sustained increase from the acute phase of infection in rapid and normal progressors (16). In these HIV-infected individuals, mBAFF expression was preferentially upregulated in blood myeloid dendritic cells (DC) (defined as HLA-DR^+^CD11c^+^) and their precursors (HLA-DR^+^CD14^+^CD11c^+^) (16). In a cohort of untreated individuals with primary HIV infection, we found that circulating BAFF levels were consistently increased at diagnosis (20–45 days after infection) but rapidly decreased toward baseline levels by 2–3 months of infection (1 month of follow-up) (19). Whereas mBAFF was mainly present in intermediate monocytes (CD14^+^CD16^+^) of healthy individuals, its expression was preferentially enhanced in CD1c^+^ DC and non-classical (CD14^lo^CD16^hi^) monocytes in individuals with primary HIV infection (19). A similar trend was observed in BDCA-3^+^ DC and intermediate monocytes but did not reach significance. *In vitro*, the virus itself can directly drive mBAFF expression and its subsequent release in monocytes as well as in monocytes-derived DC and macrophages. *In vivo*, type I and II IFN could also contribute to BAFF increase. This virus-mediated effect is essentially independent on replication since it was observed with AT2-inactivated virus. Extending our results, Gomez et al. recently showed that HIV-1 does not induce BAFF expression in monocyte-derived macrophages displaying a M1 phenotype (92). Unexpectedly, our findings showed that mBAFF was expressed by a majority of pDC in healthy individuals, an expression that strongly decreased in patients with primary HIV infection. However, this loss was not due to BAFF release since pDC are unable to cleave mBAFF (19, 38, 39). Preferential cognate interactions of pDC with MZ and memory B-cells (93, 94) might relay on mBAFF binding to TACI-short, highly expressed by these B-cell subtypes (80).

**Figure 1:**
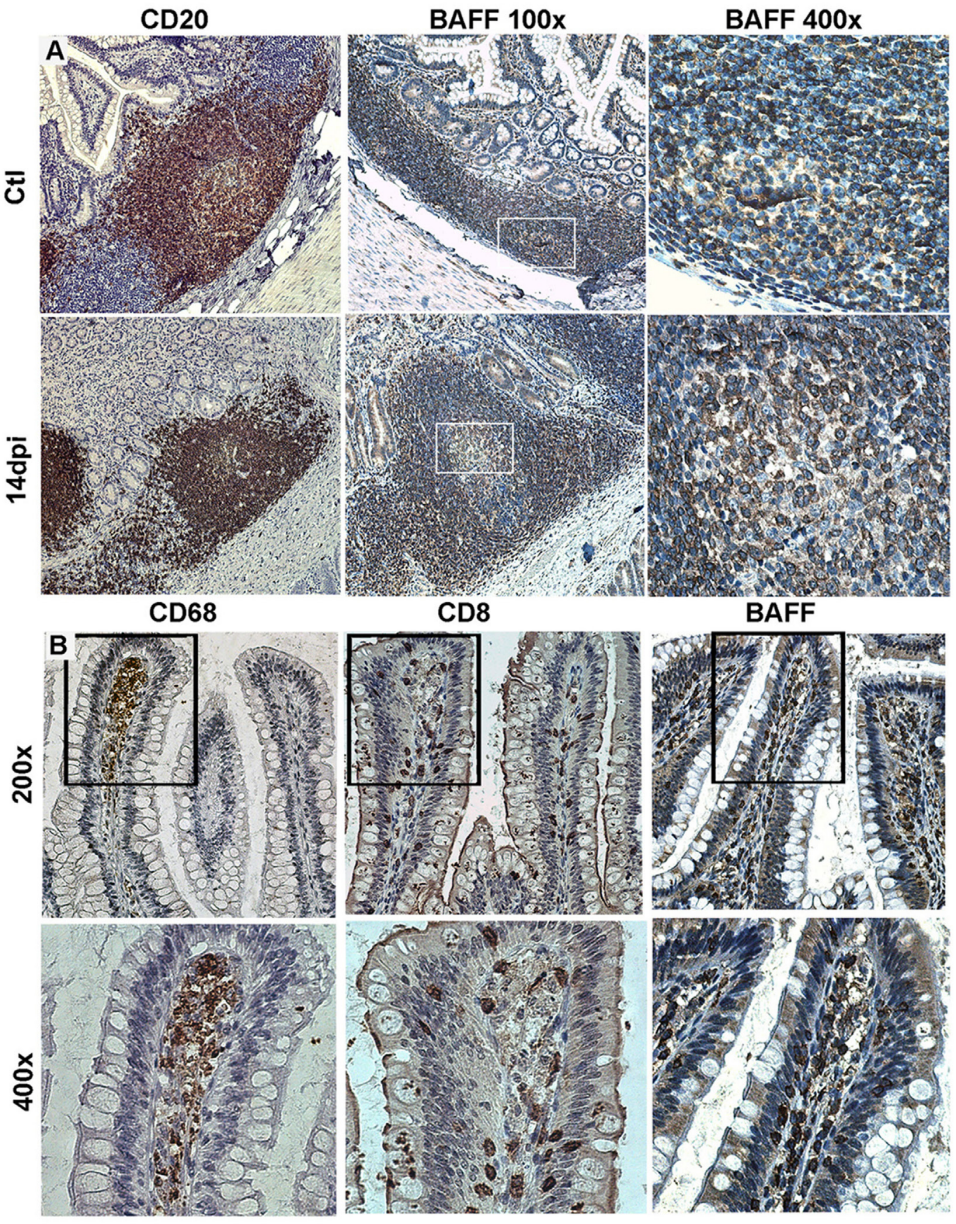
Tissue expression of B-cell-activating factor (BAFF) in SIV-infected macaques. **(A)** Terminal ileum sections from control macaques (upper panel) and macaques infected for 14 days (lower panel) were stained with anti-CD20 (B-cells, left panels) or anti-BAFF (clone Buffy 2, middle and right panels) antibodies (Abs). Original magnification: 200× for CD20, 100× and 400× for Buffy 2. **(B)** Terminal ileum sections with clear villi present were stained with CD68 (macrophages), CD8 (CD8^+^ and intraepithelial T-cells), and Buffy 2 (BAFF expression) Abs, respectively (original magnification 200×). Inserts from upper panels are shown in the lower panels (original magnification 400×). Reproduction authorized by SpringerNature.

**Figure 2:**
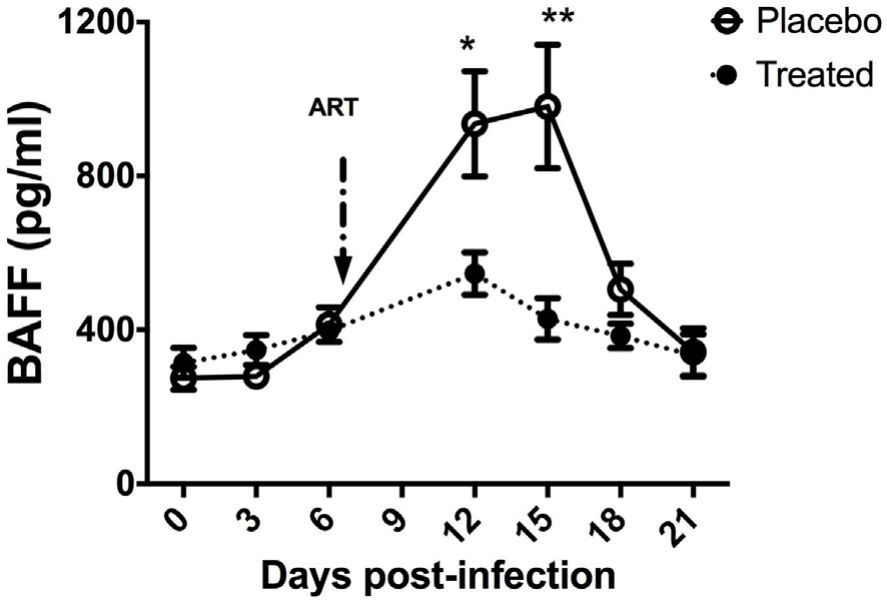
B-cell-activating factor (BAFF) levels in SIV-infected macaques upon antiretroviral therapy. Two groups of five macaques infected for 7 days by SIVmac 251 (50AID50) were treated or not (placebo) with antiretroviral therapy for 2 weeks and euthanized at day 21 post-infection (pi). Plasma BAFF concentration was determined using the BAFF Quantikine ELISA kit (R&D systems) in samples collected before infection and every 3 days pi. At each time point, mean value ± SEM is indicated for each group. At each time point, significant differences between treated and placebo groups were calculated by a Wilcoxon sign-ranked test (two-tailed, unpaired, and non-parametric *t*-test). The *p* values *(*p* < 0.05) and **(*p* < 0.01) were considered as significant.

In acutely SIV-infected macaques, we consistently observed a transient increase in BAFF plasma levels by 2 weeks of infection. BAFF levels correlate with total IgG levels, plasma viral loads and inversely with CD4 T-cell counts (21). However, steady BAFF overexpression was observed in spleen and intestinal mucosa (duodenum and terminal ileum) until 1 month post-infection. This BAFF signal was more intense in the spleen MZ, follicular mantle zone and within GC (21) but was also present all along the ileum villi in macrophages and in intraepithelial cells, likely CD8^+^ (Figure 1). According to previous data in humans, these latter cells might correspond to BAFF-expressing type-3 innate-lymphoid cells (ILC3) (95, 96). Retrospective measurement of blood BAFF levels in two groups of SIV-infected macaques treated or not by a 2-week antiretroviral therapy initiated at day 7 post-infection (97) showed a significant reduction of BAFF levels in treated animals at days 12 and 15 (42 and 56% reduction, respectively) (Figure 2). In these animals, the plasma viral load was concurrently reduced by 10^3^-fold and the proportions of memory B-cells increased in blood and spleen. Median value of plasma IgM returned to pre-infection level and SIV-specific Abs were no longer detectable after treatment (97). Thus, early initiation of antiretroviral therapy dampens BAFF increase but inhibits early virus-specific Ab production. In agreement with our data, Poudrier et al. recently showed a transient BAFF increase during the first week of SIV infection and a progressive return to baseline values after 2 months before re-increasing by 3 months post-infection (early chronic infection) in progressor animals only. These authors established that granulocytes massively contribute to BAFF production during acute and chronic phases of infection (22). This observation fits well with increased proportions of activated neutrophils in the blood of chronically HIV-infected people (98, 99). Therefore, elevated BAFF levels might constitute a good predictor of disease progression at the early chronic phase (22). This conclusion is consistent with data of comparative transcriptomic analysis showing that upregulation of *TNFSF13B* (encoding BAFF) messenger is associated with disease progression during pathogenic HIV/SIV infections (20).

In conclusion, non-classical monocytes and CD11c^+^ DC strongly contribute to elevated levels of soluble BAFF during HIV/SIV infection (16, 19, 91), but macrophages, granulocytes/neutrophils, epithelial cells, and ILC3 can also contribute to its local production in spleen and mucosa (21, 22, 100). Membrane BAFF-expressing pDC, which preferentially migrate into the vaginal mucosa and into the large intestine during pathogenic SIV infection (101, 102), might support TI B-cell response through cognate interaction with infiltrating B-cells. Through its binding to cell-type specific receptors, the virus can directly induce membrane/soluble BAFF overexpression but also the release of type I and II IFN that are keys inducers of BAFF expression. In our studies, IL1β, IL6, and TNFα are unable to modulate membrane and/or soluble BAFF overexpression by myeloid cells or pDC (19).

Preventing progression toward the chronic phase of virus infection generally requires the rapid production of potent neutralizing Abs that is rarely observed during acute HIV/SIV infection. That prompted us to interrogate the pathways of Ab production and the development of plasmablasts/cells as well as the nature of virus responsive B-cells.

## Self-Reactive B-Cells: The Last Chance for Neutralizing HIV Abs?

Whereas GC hyperplasia is one the first sign of ongoing B-cell response described in HIV-infected patients (1), the virus-specific Ab production is delayed and globally inefficient in containing virus replication and in preventing the establishment of viral reservoirs (103). Even when present, most virus-specific Abs have limited and transient capacities to neutralize the virus. Whereas pioneers studies have evidenced that inactivated purified SIV or fixed SIV-infected cells can elicit protective virus-specific Abs during infection with autologous virus (104, 105), most candidate vaccines subsequently fail to clear HIV (8). Potent bNAbs are nevertheless produced by a minority of HIV-infected individuals, generally at low titers and only after years of infection. Analyses of bNAbs that target HIV-1 envelope trimer have considerably extended our knowledge on envelope epitopes susceptible to neutralization and therefore identified new targets for vaccine trials (106). The vulnerability sites include: the membrane-proximal external region (MPER) of gp41, the CD4-binding site of gp120, an exclusively glycan epitope on the outer domain of gp120, an extended region including residues from both gp120 and gp41 between the MPER and gp120 protomers, a gp120 V2-glycan site at the apex of the envelope trimer and a gp120 V3-glycan site centered at Asn332 and the fusion peptide of HIV-1 (106, 107). Whereas passive infusion of bNAbs in humans has limited impact on HIV-1 viral loads and disease progression, two recently identified bNAbs directed against the CD4-binding site (VRC01 and 3BNC117) have significant antiviral effects (108–110). Unexpected results have shown that a subset of bNAbs concurrently recognizes nuclear or cytoplasmic human (self) antigens or proteins of commensal pathogens. These self/poly-reactive Abs preferentially recognize the CD4-binding site and the MPER region (111–114). Rare poly-reactive Abs recognizing the gp120-V3 loop have been also cloned from memory B-cells of HIV-infected patients (115, 116). bNAbs have a high degree of somatic mutation, deletions and insertions and/or elongated highly hydrophobic heavy chain complementary-determining region 3 with development of breadth correlating with acquisition of self/poly-reactivity (8, 107). Whereas these features predict negative selection, current studies reveal that ancestors of B-cells producing bNAbs are frequently self-reactive (117). A clever study recently demonstrated that breaching tolerance in mice favors the generation of cross-reactive HIV-1 self-Abs (114). Early non-neutralizing Abs directed against HIV-1 gp41 subunit are also poly-reactive (118, 119) and derive from commensal bacteria-specific memory B-cells generated in terminal ileum before infection. These B-cells acquire cross-reactivity with HIV gp41 upon T-cell driven affinity maturation, which involves GC reaction in constitutive follicles (Peyer patches or mesenteric lymph nodes) or in virus-induced isolated follicles (118, 120). Therefore, HIV might preferentially interact with self/poly-reactive B-cells in different tissues.

In physiological settings, self-reactive B-cells are eliminated at the following three major checkpoints: (i) in the bone marrow before the surface IgM-positive immature B-cell stage; (ii) in spleen MZ (or peri-follicular zone in humans) when new emigrants mature into follicular or MZ B-cells, and finally (iii) within GC during Ab affinity maturation (121). In bone marrow, 50–75% of early B-cells are self-reactive, most of which are eliminated by central tolerance mechanisms before they reach the periphery. Despite this elimination based on “tonic” BCR signaling, a substantial proportion of self/poly-reactive B-cells are still present in the blood of healthy individuals and more frequent among immature and MZ B-cells than among naïve B-cells (122, 123). Given its capacity to support the survival of transitional and MZ B-cells through BAFF-R, BAFF overproduction might abnormally rescue self-reactive B-cells as shown in murine models (124, 125) or in patients with systemic lupus erythematosus (126). If BAFF-R can directly deliver survival signal to transitional B-cells, BAFF-R signaling also interferes with BCR signaling in mice and might thus abnormally rescue early B-cells expressing self-reactive BCR (127–129). More recently, self-reactive transitional B-cells (T1 and T2) abnormally expressing TACI have been identified in BAFF transgenic mice as a consequence of BAFF excess. These TACI^hi^ transitional B-cells co-express AID (activation-induced cytidine deaminase), an enzyme mandatory for somatic hypermutation and isotype class switching, and T-bet, a transcriptional factor associated with IFNγ production and IgG class switching. Accordingly, binding of these TACI^+^ transitional B-cells by self-antigens promotes AID-mediated hyper-somatic mutations that spontaneously produce self-reactive IgG, *ex vivo* (25). Although less numerous, TACI^+^ transitional B-cells are present in wild-type mice with physiological BAFF settings. Increased proportions of T1-like (CD10^+^CD21^lo^) B-cells related to disrupted homeostasis have been reported in lymphopenic HIV-infected people with more advanced disease (67–69). Unfortunately, neither circulating BAFF level nor proportions of self-reactive B-cells, potentially HIV cross-reactive, have been estimated at the time of these studies. Whereas TACI^+^ transitional B-cells might also contribute to hypermutated Ab production during HIV infection, only rare transitional B-cells were found to express T-bet in healthy and chronically HIV-infected individuals (130). However, this might occur in highly lymphopenic HIV-infected individuals with more advanced disease.

Marginal zone B-cells that express diverse IgV_H_ genes more frequently used by self/poly-reactive Abs including by bNAbs directed against CD4bs (131), might be a “natural reservoir” for HIV cross-reactive B-cells. As mentioned earlier, human MZ B-cells highly express TACI, in particular TACI-short, and are in close contact with different BAFF/APRIL-producing cells such as macrophages, DC, neutrophils, or ILC3 in the splenic peri-follicular zone (132, 133). Thus MZ B-cells likely produce a first pool of virus-specific Abs. Indeed, we showed that the frequency of spleen MZ B-cells decreased soon after the peak of plasma viral load whereas plasmablasts/cells, mainly expressing IgG or IgM, were more numerous in the MZ 1 month post-infection in SIV-infected macaques (97). Similarly, Fontaine et al. identified a circulating population with mixed features of transitional and MZ B-cells, thought to rapidly mature into MZ B-cells upon abnormal BAFF release by myeloid cells in viremic HIV-infected people (16). Together, these data suggest that HIV induces an early differentiation of MZ B-cells into plasmablasts/cells followed by a transient lymphopenia, which tends to be compensated by accelerated repopulation of the MZ B-cell pool in patients with higher levels of replication and/or inflammation (including high BAFF levels). Studying the expansion of early self-reactive B-cells, potentially expressing T-bet, in concert with BAFF levels during pathogenic SIV/HIV infection might be valuable. Whether this repopulation favors expansion of HIV/SIV cross-reactive B-cells or their deletion remains to be studied.

## BAFF, B-Cells, and T_FH_ in GCs: From Physiological Settings to HIV/SIV Infection

Memory B-cells and long-lived plasmablasts/cells are generated within the GC through a complex process including several cycles of somatic mutations/selection as elegantly described elsewhere (134, 135). Through somatic hypermutations of V_H_ genes, an integrated process mandatory to Ab affinity maturation, the GC reaction constitutes an important stage where self-reactive B-cells are physiologically generated. Such self-reactive B-cells escaping peripheral tolerance and maturing into circulating memory IgG^+^ B-cells have been associated with autoimmunity (136) but might alternatively contribute to production of bNAbs (7). This directly questions the function of GC reaction with the generation of effectors B-cells (memory B-cells and long-lived plasmablasts/cells) in the context of chronic inflammation, where BAFF (and APRIL) can be overproduced.

Residual development of GC and efficient affinity maturation of Abs in response to TD antigen occur in BAFF or BAFF-R-deficient mice (50, 52, 53). However, GC more rapidly involute in these mice with reduced numbers of proliferating GC B-cells (centroblasts), impaired network of follicular dendritic cells (FDC) and reduced trapping of immune complexes (76). By contrast, BAFF overexpression in GC increases autoimmunity by reducing the competition between B-cell clones for T-cell help and survival signals, at least in mice (124, 137). Fibroblastic reticular cells throughout the body and FDC in GC are the main sources of BAFF in homeostatic settings (138) but also of CXCL13, which attracts B-cells to build B-cell follicles (139). During a TD response, T_FH_ constitute not only the major source of BAFF, mandatory for the B-cell survival and the selection of high affinity B-cell clones (79) but also of CXCL13 as shown in vaccinated mice and primates (140). Consistent with ongoing TD response in HIV-infected patients, high blood CXCL13 levels have been reported with concomitant decrease of CXCR5 expression by circulating B-cells (141). Whether this decrease is due to B-cell activation or to an enhanced BAFF-mediated internalization of CXCR5, it likely perturbs the recruitment of B-cells into follicles. Given that BAFF enhances the CXCL13-mediated chemotactic response of CD27^+^ human B-cells, *in vitro* (142), it could potentiate the entry of recently antigen-activated B-cells (GC founders) or of memory B-cells into the follicle during a first or second exposure to antigen/pathogen, respectively. Within GC, BAFF overproduction might either increase the CXCL13-mediated response of B-clones in the light zone or accelerate the internalization of CXCR5 in centrocytes (light zone B-cells), favoring their rapid return to the dark zone. In both case, the asymmetric recycling of B-cell clones between dark and light zones and the selection process would be impaired leading to an abnormal pattern of mutation/selection of B-cell clones required for affinity maturation (143, 144). Alternatively, BAFF excess in GC might enhance BAFF-mediated BAFF-R cleavage on TACI^+^ GC B-cells (centrocytes). Decreased BAFF-R expression might consequently abolish BAFF effect on the CXCL13 chemotactic response of centrocytes or decrease the survival of high-affinity B-cell clones (26). At which step HIV cross-reactive B-cells clones appear and why they are not eliminated as self-reactive B-cells in the context of high amount of viral antigens is far from being clear. Vulnerability sites frequently buried in the envelope structure or masked by glycans are probably weakly accessible. This might favor their ignorance during the GC selection process.

**Figure 3:**
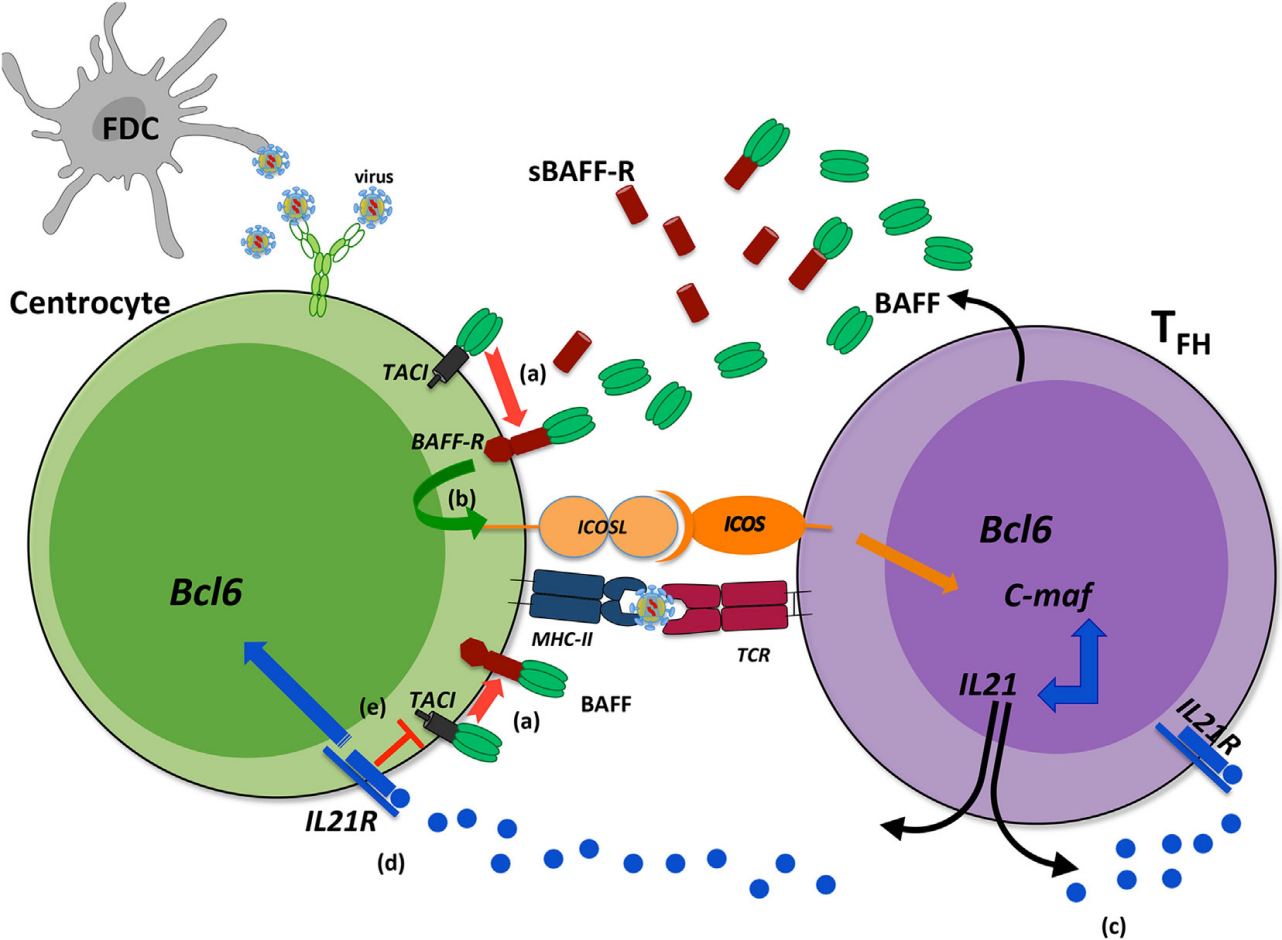
Transmembrane activator and CAML interactor (TACI) as a key regulator of B-cell-activating factor (BAFF)-dependent BAFF-R cleavage in germinal center (GC). During a T-dependent response, follicular helper T-cells (T_FH_) produce BAFF that can bind either to BAFF-R or to TACI. When BAFF is locally released in excess, its binding to BAFF-R can induce the cleavage of BAFF-R in a TACI-dependent manner from the surface of centrocytes (a). Reduced BAFF-R signaling leads to decreased ICOSL expression on B-cells (b) and therefore dampens ICOS signal, mandatory for T_FH_ maintaining and IL21 production. This might constitute a physiological regulatory mechanism, exacerbated when high amounts of antigens are maintained within GC. IL21 is a key cytokine for T_FH_ that ensures their survival (c) and that of light zone GC B-cells (d). In addition, IL21 decreases TACI expression that might prevent early TACI-dependent BAFF-R cleavage (e). Such regulatory roles would imply that IL21 and BAFF are produced sequentially during the GC reaction with possible consequences on recycling and differentiation of GC B-cells.

In addition to its action on GC B-cells, two independent studies implied TACI in T_FH_ expansion (Figure 3). In the first study, Ou et al. showed that BAFF preferentially binds BAFF-R and upregulates ICOSL expression by GC B-cells in physiological settings. When it is locally overproduced, BAFF also binds to TACI on GC B-cells leading to down modulation of BAFF-R-mediated ICOSL expression and subsequently T_FH_ expansion (78). According to recent data on BAFF-R processing (26), one hypothesis could be that BAFF excess leads to TACI-mediated BAFF-R cleavage, which reduces BAFF-R signaling and thus down-modulates ICOSL expression. In a second study, IL21 produced by T_FH_, besides supporting the survival of both T_FH_ and GC B-cells through IL21R, also diminishes TACI expression thus preventing premature loss of T_FH_ (79). As T_FH_ concurrently produce BAFF and IL21, a delicate balance is thought to control efficient GC reaction. The existence of two human TACI isoforms could further complicate our understanding of the role of BAFF and its receptors, BAFF-R and TACI, in GC reaction.

Impaired helper functions of T_FH_ at the chronic phase of HIV/SIV infection (2, 6, 145) likely contribute to inefficient B-cell response to HIV/SIV. However, early functional T_FH_ are present at elevated frequencies in nodal GC from the acute phase of HIV infection and their presence correlates with the breadth of bNAbs at the chronic phase (146). Thus, generation of bNAbs is dependent on the preservation of T_FH_ functions, likely impaired in CXCR3^+^ T_FH_ (147). As recently shown, human T_FH_ express BAFF-R and release more IFNγ after culture with BAFF (148), thus BAFF excess might contribute to T_FH1_-expansion during HIV/SIV infection. Being produced by FDC and T_FH_ in GC, BAFF likely exerts a physiological role on T_FH_, during response to TD natural or vaccine antigens. In conclusion, the overexpression of BAFF might impair GC reaction and even modulate T_FH_ functions.

## Memory B-Cells: The Weak Link in HIV/SIV Infection

It is now well established that chronically HIV-infected patients have an impaired memory B-cell compartment with lower frequency of HIV-specific and vaccine-specific memory B-cells as well as reduced anti-vaccine Abs (149–151). In addition to lower proportions of memory B-cells, viremic HIV-infected individuals also exhibited increased proportions of CD21^lo^ mature B-cells (68, 81). This subset highly expressed BCMA and TACI but had decreased BAFF-R expression and BAFF binding. Based on this phenotype profile and on the concomitant increase in CD27, CD38, and CXCR3 expression, these CD21^lo^ B-cells were first considered as circulating plasmablasts, prone to apoptosis and Ab production (67, 81) and expanded as a consequence of HIV-induced hyperactivation. After the identification of a subset of CD20^hi^CD21^lo^ tissue memory cells in human tonsils exhibiting signs of exhaustion (82), the classification of this CD21^lo^ population in HIV-infected patients has been revised. In addition to plasmablasts, the CD21^lo^ B-cell subset comprised CD27^+^CD21^lo^ and CD27^lo^CD21^lo^ cells often referred to as activated memory (ActM) and tissue-like memory (TLM) B-cells, respectively. These subsets differ from conventional RM B-cells by their expression of activation, inhibitory and/or apoptotic markers (69). In healthy donors, RM B-cells constitute the predominant fraction of blood memory B-cells with low percentages of CD21^lo^ memory B-cells (152). By contrast, ActM and TLM are overrepresented in blood of chronically HIV-infected patients (153) and in rapidly progressing SIV-infected macaques (154). In contrast to influenza or tetanus-specific Abs enriched in RM B-cells, HIV-specific Abs are enriched in TLM B-cells in untreated individuals (155, 156). More recently, Muema et al. reported increased proportions of ActM, TLM, and plasmablasts but decreased proportions of naïve B-cells in vertically HIV-infected children in a viral-load-dependent manner (83). In agreement with other studies in children, lower IgG levels and proportions of switched memory B-cells against childhood vaccines were observed (150, 157, 158). In these HIV-infected children, circulating BAFF levels were elevated whereas BAFF-R and TACI expression were respectively decreased and increased in most B-cell subsets. B-cell interaction with viral proteins that can induce BCR- or TLR-mediated B-cell activation (9, 10, 159) might also increase TACI expression, possibly stabilized at the membrane by BAFF binding as shown in mice (79). By contrast, decreased BAFF-R expression might be due to potent receptor internalization in the presence of high BAFF levels as suggested during malaria infection (17, 160) or to enhanced BAFF-mediated BAFF-R processing (26).

It is not clear whether increase in TACI expression has any influence on ActM or TLM functions or survival, *in vivo*. Survival of human and simian RM B-cells (BAFF-R^+^ TACI^hi^) is less dependent on BAFF than that of naïve and transitional B-cells in physiological settings (161, 162). Moreover, BAFF levels correlate with proportions of MZ and RM B-cells, but not of CD21^lo^ memory B-cells in HIV-infected children (83). By contrast, BAFF levels and proportions of CD21^lo^ memory B-cells were concomitantly increased in individuals infected by *Plasmodium* (17, 160), an infection setting where the frequencies of TLM-like B-cells are increased (18, 163).

Similar to TLR9 ligands that elicit responses in TLM-like B-cells of malaria-exposed people (18, 164), BAFF/APRIL as TACI ligands might deliver differentiation signals to these B-cells through TACI and its downstream TLR-like signaling pathways (87) Whereas overrepresentation of CD21^lo^ memory B-cells is consistently associated with chronic inflammation, the mechanisms leading to this phenotype are largely unknown. Studies in mice and more recently in HIV-infected people showing T-bet expression by CD21^lo^ B-cells suggest simultaneous actions of pathogen-derived nucleic acids, through TLR9/7, and Th1-cytokines (IFNγ) (130, 165–167). According to high TACI expression in HIV-infected children, BAFF overexpression might directly or indirectly (for example, by upregulating IFNγ production by NK or Th1-cells) contribute to the generation or survival of these CD21^lo^ memory B-cells and thus Ab-mediated protection against HIV-1.

## Conclusion

B-cell-activating factor and its receptors (BAFF-R, TACI, and BCMA) are key actors for the B-cell survival and the immune responses of mature B-cells. Whereas BAFF-R is more widely expressed throughout the B-cell differentiation, TACI now appears as a key regulator of various BAFF-mediated responses. Indeed TACI is spontaneously released upon activation and orchestrates the cleavage of BAFF-R-BAFF complexes. This might have a major impact on memory and MZ B-cells that are TACI^hi^. Perturbations of these regulatory mechanisms likely impair the GC reaction: GC B-cell selection/survival or recycling between dark and light zones as well as the generation of appropriate effector B-cells during TD responses. Soluble but also membrane BAFF overexpression by key DC subsets during HIV/SIV infection might subsequently initiate the unexpected expansion of HIV cross-reactive B-cell clones and atypical memory B-cells. In this review, we pointed out previous data arguing for the involvement of BAFF in HIV-mediated B-cell dysfunctions and discussed more recent data on BAFF and TACI in physiological settings. Accordingly, we suggest BAFF-mediated mechanisms that could modulate B-cell response during pathogenic HIV/SIV infection. Our research around BAFF is part of a more global approach that aims to identify B-cell subset(s), which could constitute a reservoir of HIV cross-reactive B-cells, and to understand how to promote their expansion and/or prevent their elimination. This information is likely a prerequisite for the development of next-generation HIV vaccines.

## Author Contributions

All authors contribute to the writing of this review.

## Conflict of Interest Statement

The authors declare that the research was conducted in the absence of any commercial or financial relationships that could be construed as a potential conflict of interest.
